# The Experimental Process Design of Artificial Lightweight Aggregates Using an Orthogonal Array Table and Analysis by Machine Learning

**DOI:** 10.3390/ma13235570

**Published:** 2020-12-07

**Authors:** Young Min Wie, Ki Gang Lee, Kang Hyuck Lee, Taehoon Ko, Kang Hoon Lee

**Affiliations:** 1 Department of Materials Engineering, Kyonggi University, Suwon 16227, Korea; supreme98@kyonggi.ac.kr (Y.M.W.); gglee@kyonggi.ac.kr (K.G.L.); 2 Center for Built Environment, Sungkyunkwan University, Suwon 16419, Korea; diappong@hanyang.ac.kr; 3 Department of Medical Informatics, The Catholic University of Korea, Seoul 06591, Korea; thko@catholic.ac.kr; 4 Department Civil & Environmental Engineering, Hanyang University, Seoul 04763, Korea

**Keywords:** orthogonal array experiment design method, lightweight aggregate, support vector regression, machine learning, sintering process

## Abstract

The purpose of this study is to experimentally design the drying, calcination, and sintering processes of artificial lightweight aggregates through the orthogonal array, to expand the data using the results, and to model the manufacturing process of lightweight aggregates through machine-learning techniques. The experimental design of the process consisted of L_18_(3^6^6^1^), which means that 3^6^ × 6^1^ data can be obtained in 18 experiments using an orthogonal array design. After the experiment, the data were expanded to 486 instances and trained by several machine-learning techniques such as linear regression, random forest, and support vector regression (SVR). We evaluated the predictive performance of machine-learning models by comparing predicted and actual values. As a result, the SVR showed the best performance for predicting measured values. This model also worked well for predictions of untested cases.

## 1. Introduction

Aggregates are the second most commonly consumed raw material by humanity after water. Therefore, the aggregate industry requires a highly strategic approach in terms of the supply of raw materials for construction and to protect the environment. [1] According to an article in the Korea Construction Newspaper, in 2019 the amount of aggregate used in Korea stood at approximately 200 million tons per year, half of which is mined directly [2]. This presents a major environmental burden and leads to the depletion of natural aggregate resources. Accordingly, in recent years, research focusing on artificial lightweight aggregates has been actively conducted from the viewpoint of environmental protection and waste recycling. Various wastes have been studied, including coal fly ash [3,4], polymer waste [5], sewage sludge [6,7], and waste glass [8,9]. However, these studies focus on the waste itself rather than the manufacturing process of aggregates, and there are relatively few studies on the manufacturing process of lightweight aggregates.

A few manufacturing methods for artificial lightweight aggregate have been considered, with most common being firing in a rotary kiln using natural materials such as expanded clay [10,11]. Numerous studies of firing processes for artificial lightweight aggregates have been conducted. In laboratory-scale research, methods such as rapid sintering [12,13], two-stage sintering [14], and normal sintering are most commonly reported [15,16]. Each experimental result differs slightly depending on conditions, but it was commonly noted that the calcination section must be shortened to form internal pressure. The importance of internal pressure and viscous behavior for the bloating of artificial lightweight aggregates has recently attracted attention. According to Dondi et al. [17], the foaming properties of an aggregate should be explained not only in terms of the chemical components used but also in terms of the internal pressure and viscous behavior. In a study by Moreno-Maroto et al. [18], viscous behavior was found to be very important for the bloating of aggregates, and only a very small amount of gas is involved in bloating. Moreover, in work by Wie et al. [19], an orthogonal array experimental design was utilized to find the optimal conditions for the bloating activation of aggregates under a normal sintering condition. It was shown that for the bloating of an aggregate, the calcination section should be short and the proper time should be maintained in the calcination section for viscous behavior. This is in line with existing theory [17]. In addition, a prediction of the physical properties using an orthogonal array experiment design was made, and the predicted value was found to be relatively suitable. However, although it was possible to find the optimal conditions and make local predictions under certain experimental conditions using the orthogonal array experiment design, it was difficult to predict conditions that were not tested at all within the experimental range. Additionally, the tendency of the experimental results was not defined uniformly. Therefore, additional techniques are needed to predict untested parts rationally and to define trends clearly according to process changes.

Currently, modeling techniques using machine learning are used in various fields, such as agriculture [20], biology [21], chemistry [22,23], and climate prediction [24]. Regarding construction materials, many studies have been conducted to predict the physical properties of concrete [25,26,27,28,29,30]. However, these studies require more than 200 sizable instances of data from the literature, making it very difficult to obtain with a typical experiment. For this reason, most studies rely on modeling via the collection of other studies. This is an advantageous way to define an individual variable academically and create an experimental formula. However, this method is not practical to be applied directly to industrial sites because it is difficult to simultaneously apply various variables of industrial sites and individual conditions of all sites cannot be considered. In order to overcome these limitations and create a model that can be practically utilized in the industrial field, it is necessary to model using reliable data under relatively controlled conditions with various variables. The experimental design method using an orthogonal array table is very practical because it can expand a large amount of data with relatively few experiments and, through this, influences on a wide variety of variables can be simultaneously applied. In addition, data expansion by an orthogonal array table can provide a sufficient amount of data required for modeling. In addition, since the machine-learning technique analyzes data with a computer and presents the optimal predicted value, it is expected that the use of the two techniques mentioned above will be very practical in the industrial field.

Therefore, in this study, in order to model the firing process of the artificial lightweight aggregates, data were expanded by the orthogonal array table, and modeled using machine-learning techniques by the expanded data. The applied techniques are linear regression, random forest, and support vector regression techniques, and these are the most used techniques in analysis through machine learning. The suitability of the models was evaluated according to each evaluation index, and the predicted result through the model and the actual test result were compared and analyzed. By verifying the three models and comparing them with actual results, we confirmed the effectiveness of data expansion using an orthogonal array table experimental design, and reviewed the most suitable machine-learning techniques for this.

## 2. Experiment Method

### 2.1. Experimental Variables and Experimental Design

In order to separate the variables according to the unit process of lightweight aggregate and to facilitate data expansion, the experiment was designed as follows. In the experiment, the unit process was defined for each temperature section. The drying process is defined as the room temperature (r.t.) ~600 °C section. The drying section was divided into r.t. ~300 °C and 300~600 °C again, to distinguish between the evaporation section of water and the section where the combustion of organic matter begins. The calcination process is defined as the 600 °C~bloating activation temperature section, and the bloating activation temperature soaking section is defined as the bloating start and activation section. Table 1 shows the experimental variables. The design utilized an orthogonal array experiment table with the indicated variables. After conducting the designed experiment, the density of the aggregates produced was measured according to KS F 2503 [31].

### 2.2. Data Expansion by Means of an Orthogonal Array Experiment Design

Full factor design requires a lot of experimentation because in real industry it is usually composed of a large number of factors. The method of selecting a limited number of experiments is called a partial factor experiment, and although this method is widely known, general guidelines for application and analysis have not been established. The orthogonal array table approach drastically reduces the amount of experimentation compared to the full factor experimental design, and the use of data is relatively well known [32,33,34,35,36]. In this study, the number of experiments was significantly reduced by using an orthogonal array table, and data comparable to full factor experiments can be obtained through data expansion. Recently, a research method combining an orthogonal order table and various analysis methods has been widely used. In many cases, it was found that the data obtained from the orthogonal array table and the results obtained from the full factor experiment were not significantly different [37,38,39,40]. 

In order to investigate the possibility that the property prediction by the orthogonal array technique will be used in the study, the predicted value of the density based on the results of the orthogonal array experiment was obtained. The prediction for the experiment was conducted using Minitab (Minitab1.9, Minitab Inc., Pennsylvania, PA, USA). The prediction of the orthogonal array test is a result reflecting the increase or decrease when the variables (*D_i_*, *D_j_*, *D_k_*, *D_l_*) desired by the experimenter are compared to the total average value (*D_tot_*_._). This proceeds as follows (Equation (1)):

(1) $$D_{pred.}=D_{tot.}+\left(D_{tot.}-D_{i}\right)+\left(D_{tot.}-D_{j}\right)+\left(D_{tot.}-D_{k}\right)+\left(D_{tot.}-D_{l}\right)$$

(*D_pred._*: Predicted density value, *D_tot._*: Total average density value, *D_i,j,k,l_*: Average value for each variable)

In this experiment, 18 experiments were expanded to 486 by designing an experiment with an orthogonal array table L_18_ (3^6^6^1^). This is the same as the number of full factors of the designed experiment, and the planned experiment is expanded to 3^4^ × 6^1^. This data expansion is possible because the effects of individual variables can be separated in an experiment designed by an orthogonal array table. Because of this characteristic, experiments using an orthogonal sequence table are widely used in the industry [41,42,43].

### 2.3. Machine-Learning Analysis Using Extended Data

**Figure 1:**
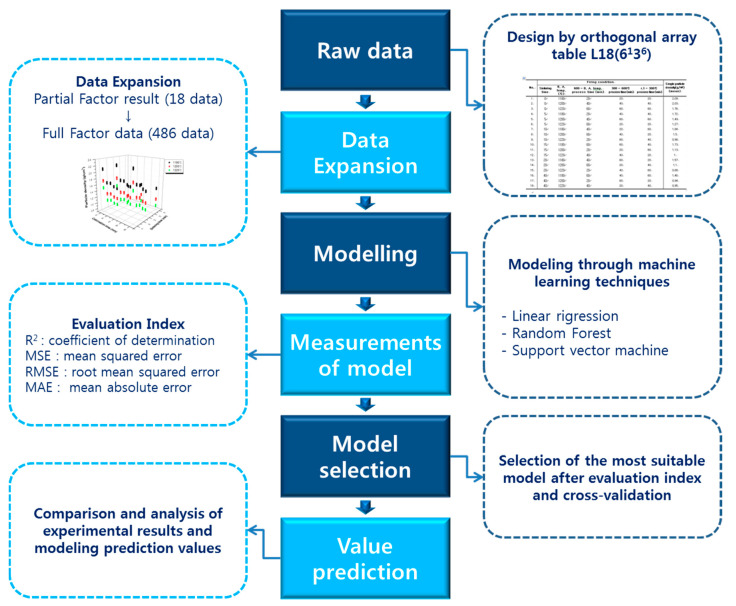
Flow chart for data expansion and machine-learning analysis.

Figure 1 shows the flow of analysis using extended data using an orthogonal array table. Extended data are trained on three regression models: linear regression, random forest, and supporter vector regression (SVR), while cross-validation is performed using the leave-one-out cross-validation technique. After that, the model’s fit and predictive performance are determined through four evaluation indicators to find the optimal model. Then, the predicted particle density values obtained through the selected optimal regression model were compared with the experimental values to confirm the effectiveness of the machine-learning technique.

## 3. Machine-Learning Regression Methods

Regression analysis is a set of methods for estimating the relationship between a continuous dependent variable and several independent variables. The functional relationship between the dependent variable Y and the independent variables $X_{1},\;X_{2},\ldots ,\;X_{p}$ is expressed as the following Equation (2):

(2) $$Y=f\left(X_{1},X_{2},\ldots ,\;X_{p}\right)+\varepsilon$$

where *ε* is called residual, the difference between the observed value of Y and the predicted value. Regression methods try to find the functional relationship f in the data. 

### 3.1. Linear Regression

For the given data ${\left\{y_{i},x_{i1},x_{i2},\cdots x_{ip}\right\}}_{i=1}^{n}$, the linear regression discovers the linear relationship as the following Equation (3):

(3) $$y_{i}=\beta_{0}+\beta_{1}x_{i1}+\beta_{2}x_{i2}+\cdots +\beta_{p}x_{ip}+\varepsilon_{i}\;\left(\mathrm{for}\;i=1,\cdots ,n\right)$$

The regression coefficients $\beta_{0},\beta_{1},\cdots ,\beta_{p}$ can be estimated using the ordinary least-squares (OLS). The least-squares estimate ${\hat{\beta }}_{0},{\hat{\beta }}_{1},\cdots ,{\hat{\beta }}_{p}$ is defined as the value that minimizes the following Equation (4).

(4) $$\overset{n}{\underset{i=1}{\sum }}\varepsilon_{i}^{2}=\overset{n}{\underset{i=1}{\sum }}{\left\{y_{i}-\left(\beta_{0}+\beta_{1}x_{i1}+\beta_{2}x_{i2}+\cdots +\beta_{p}x_{ip}\right)\right\}}^{2}$$

Therefore, the predicted value of the dependent variable y for the new independent variables $x_{1},x_{2},\cdots ,x_{p}$ can be obtained through the following Equation (5).

(5) $$\hat{y}={\hat{\beta }}_{0}+{\hat{\beta }}_{1}x_{1}+\cdots +{\hat{\beta }}_{p}x_{p}$$

### 3.2. Regression Tree

The regression tree is a regression version of decision tree, which is one of the most popular machine learning algorithms. In the process of regression tree training, the entire area of the given data ${\left\{y_{i},x_{i1},x_{i2},\cdots ,x_{ip}\right\}}_{i=1}^{n}$ is divided into M areas $R_{1},R_{2},\cdots ,R_{M}$ and the constant value $c_{m}$ is predicted in each area. The tree model that does this is shown below (Equation (6)):

(6) $$f\left(x\right)=\overset{M}{\underset{m=1}{\sum }}c_{m}I\left(x\in R_{m}\right).$$

The values of $c_{m}$ and $R_{m}$ are determined based on the impurity level, and the measurement is the sum of the squared errors $Q_{m}\left(T\right)=\sum_{i=1}^{n}{\left(y_{i}-f\left(x_{i}\right)\right)}^{2}$. If the given split variable $x_{j}$ is continuous, then if the split point is s, $R_{1}\left(j,s\right)=\left\{x:x_{j}\leq s\right\}$ and $R_{2}\left(j,s\right)=\left\{x:x_{j}>s\right\}$ can be defined. Here, we find the following optimal solution for separation criteria (Equation (7)):

(7) $$\underset{j,s}{\min}\left(\underset{c1}{\min}\underset{x_{i}\in R_{1}\left(j,s\right)}{\sum }{\left(y_{i}-c_{1}\right)}^{2}+\underset{c2}{\min}\underset{x_{i}\in R_{2}\left(j,s\right)}{\sum }{\left(y_{i}-c_{2}\right)}^{2}\right)$$

The corresponding solutions having the minimum value for the given j and s values are ${\hat{c}}_{1}$ and ${\hat{c}}_{2}$, which are given as the average $y_{i}$ value of the data belonging to $R_{1}\left(j,s\right)$ and $R_{2}\left(j,s\right)$, respectively. After finding the optimal separation criterion (j,s) through a proper optimization technique, the same process is repeated for both areas. If the new data $x\in R_{m}$, we predict $\hat{y}={\hat{c}}_{m}$.

### 3.3. Random Forest

The random forest developed by Breiman [44] takes into account the large variance of decision trees and is a model that uses an ensemble-learning method to learn multiple decision trees randomly. An ensemble is a method that improves prediction accuracy levels by learning multiple models from given data and then synthesizing the prediction results of multiple models.

**Figure 2:**
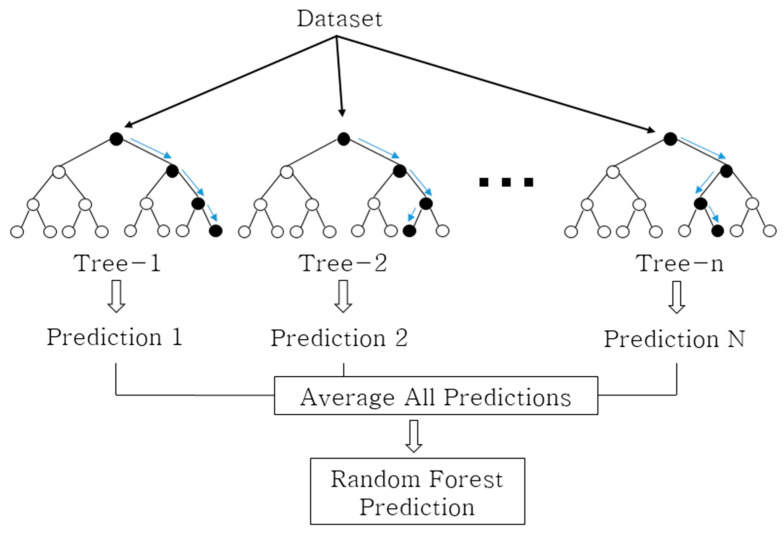
Schematic diagram of the random forest algorithm.

The random forest algorithm is shown below (Figure 2):

1. Create bootstrap sample $L^{*}={\left\{y_{i}^{*},x_{i}^{*}\right\}}_{i=1}^{n}$ using *n* data for training data $L={\left\{y_{i},x_{i}\right\}}_{i=1}^{n},\;x_{i}\in R^{p}$.
2. At $L^{*}$, only k(≪p) values of independent variables are randomly selected to generate a decision tree.
3. Compute the final predicted value of the random forest by combining the predicted values of the trees. In general, the average value is used.

### 3.4. SVR (Support Vector Regression)

Support vector regression (SVR) is a regression version of support vector machine. The purpose of SVR is to find the optimal hyperplane for fitting data instances well. Given the data $L={\left\{y_{I},x_{i}\right\}}_{i=1}^{n},\;x_{i}\in R^{m}$, the function of the SVR model proceeds according to Equation (8).

(8) $$f\left(x\right)=w^{T}\cdot x+b,\;w\in R^{m},\;b\in R$$

This process learns by adding extra variables $\xi ,\xi^{*}$ that allow errors. The objective function in Equation (9) is optimized so that the norm function of w is minimized while the deviation between the actual data and the predicted data is within ε.

(9) $$\underset{w,b,\xi_{i},\xi_{i}^{*}}{\min}\left(\frac{1}{2}{||w||}^{2}+C\overset{n}{\underset{i=1}{\sum }}\left(\xi +\xi^{*}\right)\right).$$

At this time, the constraint condition is expressed by Equation (10).

$$y_{i}-wx_{i}-b\leq \varepsilon +\xi_{i}$$

$$b+wx_{i}-y_{i}\leq \varepsilon +\xi_{i}^{*}$$

(10) $$\xi_{i},\xi_{i}^{*}\geq 0,\;\;\;i=1,2,\cdots ,n$$

The Equation for optimizing the objective function can be solved simply via a dual problem using the Lagrange multiplier and can be expressed as Equation (11) with the Lagrange multiplier.

$$L=C\overset{n}{\underset{i=1}{\sum }}\left(\xi_{i}+\xi_{i}^{*}\right)+\frac{1}{2}{||w||}^{2}$$

(11) $$-\overset{n}{\underset{i=1}{\sum }}\left(\eta_{i}\xi_{i}+\eta_{i}^{*}\xi_{i}^{*}\right)-\overset{n}{\underset{i=n}{\sum }}\alpha_{i}\left(\varepsilon +\xi_{i}-y_{i}+wx_{i}+b\right)-\overset{n}{\underset{i=1}{\sum }}\alpha_{i}^{*}\left(\varepsilon +\xi_{i}^{*}+y_{i}-wx_{i}-b\right).$$

L denotes Lagrangian function and $\alpha_{i},\alpha_{i}^{*},\eta_{i},\eta_{i}^{*}$ are called Lagrangian multipliers; for L, the differential value of each parameter can be expressed as Equation (12):

$$\frac{\partial L}{\partial w}=0\;\to \;w-\sum_{i=1}^{n}\left(\alpha_{i}-\alpha_{i}^{*}\right)x_{i}=0$$

(12) $$\frac{\partial L}{\partial \xi_{i}}=0\;\to \;c-\alpha_{i}-\eta_{i}=0$$

Therefore, the objective function of SVM is as expressed as Equation (13).

(13) $$\underset{\alpha_{i},\alpha_{i}^{*}}{\min}\left(\overset{n}{\underset{i=1}{\sum }}\left(\alpha_{i}-\alpha_{i}^{*}\right)y_{i}-\varepsilon \overset{n}{\underset{i=1}{\sum }}\left(\alpha_{i}+\alpha_{i}^{*}\right)-\frac{1}{2}\overset{n}{\underset{i=1}{\sum }}\overset{n}{\underset{j=1}{\sum }}\left(\alpha_{i}-\alpha_{i}^{*}\right)\left(\alpha_{j}-\alpha_{j}^{*}\right)x_{i}^{T}x_{j}\right).$$

The Lagrangian function L can be expressed as $\alpha_{i},\alpha_{i}^{*}$, and the final prediction model can be obtained using the differential value for w. (Equation (14))

$$f\left(x\right)=w^{T}\cdot x+b=\overset{n}{\underset{i=1}{\sum }}\left(\alpha_{i}-\alpha_{i}^{*}\right)x_{i}^{T}x+b.$$

$$b=y_{i}-\varepsilon -\overset{n}{\underset{i=1}{\sum }}\left(\alpha_{i}-\alpha_{i}^{*}\right)x_{i}^{T}x.$$

(14) $$f\left(x\right)=w^{T}\cdot x+b=\sum_{i=1}^{n}\left(\alpha_{i}-\alpha_{i}^{*}\right)x_{i}^{T}x+b$$

**Figure 3:**
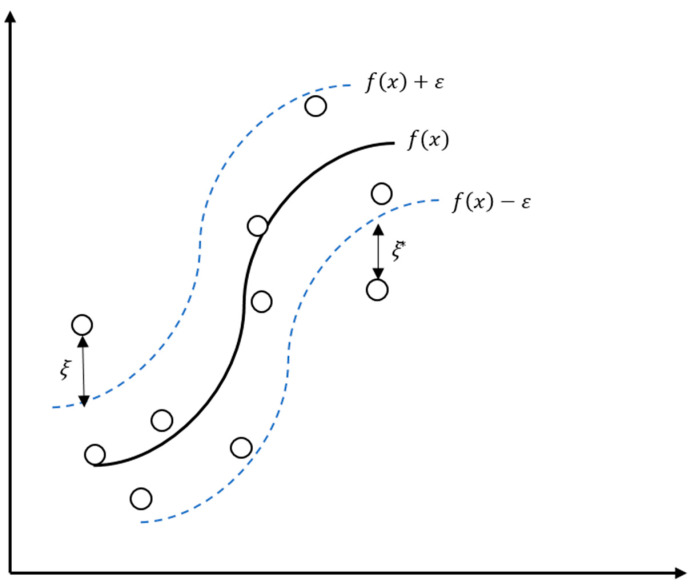
Schematic diagram of non-linear support vector regression.

SVR described above is a linear prediction technique. In order to find a non-linear function as shown in Figure 3, it is necessary to map the given data into a high-dimensional feature space, called kernel space. Several kernel functions are available [45], but the radial basis function (RBF) kernel function is used in this study (Equation (15)):

(15) $$k\left(x_{i},x_{j}\right)=\exp\left(-\gamma |\left|x_{i}-x_{j}\right|{|}^{2}\right).$$

Therefore, the SVR prediction function, as in Equation (16), can be obtained:

(16) $$f\left(x\right)=\sum_{i=1}^{n}\left(\alpha_{i}-\alpha_{i}^{*}\right)k\left(x_{i},x_{j}\right)+b$$

## 4. Evaluation of the Performance

### 4.1. Leave-One-Out Cross Validation, (LOOCV)

In general, when evaluating the performance of a predictive model, the model is applied to an unseen test set. In contrast, when using cross-validation, data are divided into several non-overlapping groups, and the model is created by changing the dataset used for training and verification. Cross-validation is widely used as a model evaluation method to compensate for the poor predictability when introducing new data, as the model is overfit to the data used for fitting in machine learning. Leave-one-out cross-validation is a viable means of comparing a value predicted by a model and an actual value with respect to the remaining data after setting the model using the remaining data, apart from one data instance out of the entire dataset. This method is suitable when the number of instances is small.

### 4.2. Performance Evaluation Measures

For an evaluation of the three models used in this paper, the coefficient of determination ($R^{2}$), the mean squared error (MSE), the root mean squared error (RMSE), and the mean absolute error (MAE) are used.

The $R^{2}$ is a measure of the degree to which the estimated linear model fits the given data and shows the explanatory power of the estimated linear model. The $R^{2}$ has a value of 1 or less, and it can be said that the closer to 1 the value is, the better the explanatory power of the model becomes. $y_{i}$ is the actual target value of the i-th observation, $\bar{y}=\frac{1}{n}\sum_{i=1}^{n}y_{i}$ is the average of n target values, and ${\hat{y}}_{i}$ is the target value of the *i*-th observation predicted by the regression model. The $R^{2}$ is expressed by the following Equation (17).

(17) $$R^{2}=\;1-\frac{\sum_{i=1}^{n}{\left(y_{i}-{\hat{y}}_{i}\right)}^{2}}{\sum_{i=1}^{n}{\left(y_{i}-{\bar{y}}_{i}\right)}^{2}}.i=1,\cdots ,n$$

The MSE can rank the performances of several models. The average error value is within the range of [0,∞], and the smaller this value is, the better the model performance becomes. Equation (18) for the MSE is as follows:

(18) $$mean\;squared\;error=\;\frac{\sum_{i=1}^{n}{\left(y_{i}-{\hat{y}}_{i}\right)}^{2}}{n}.$$

The MSE can effectively rank models, but the difference between the actual and predicted values of the dependent variable is unknown. The RMSE can be used to make a more meaningful interpretation of the model’s prediction error. (Equation (19))

(19) $$\mathrm{root}\;mean\;squared\;error=\sqrt{\frac{\sum_{i=1}^{n}{\left(y_{i}-{\hat{y}}_{i}\right)}^{2}}{n}}.$$

Given that the RMSE contains a square term, it tends to overestimate the error because large errors become emphasized. The MAE can be used as an alternative to this situation (Equation (20)).

(20) $$mean\;absolute\;error=\frac{\sum_{i=1}^{n}\left|y_{i}-{\hat{y}}_{i}\right|}{n}.$$

## 5. Results and Discussion

### 5.1. Property Prediction and Data Expansion by the Orthogonal Array Experiment Design

**Figure 4:**
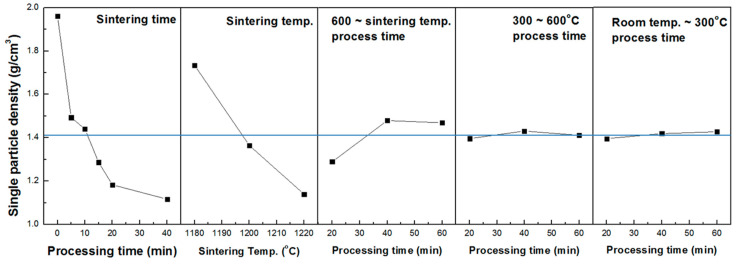
Analysis result of experiment designed with orthogonal array table.

Table 2 shows the orthogonal array experiment design table and single particle density measurement results. The experiment was designed and tested with an orthogonal array table L_18_(3^6^6^1^). The three important variables in the experiment were sintering temperature, sintering time, and calcination time. Drying conditions did not significantly affect the properties of the final aggregates. Figure 4 shows the effect of each variable. It can be seen that the higher the sintering temperature, the lower the density of the aggregate. Moreover, the longer the holding time, the lower the density gradually, but after 20 min, the density no longer decreased. In addition, when the calcination time was 20 min, a lower density level could be obtained.

According to Kõse et al. [46], the generation and development of pores can be divided into three stages.

(1) Gas is generated inside the aggregates.
(2) The generated gas creates pressure, the pore walls are destroyed by the pressure, and the pores merge.
(3) The pores grow due to the pressure difference between small pores and large pores.

At this time, in the initial stage, the processes of (1) and (2) take place, afterwards, it has been found that the process of (3) becomes dominant. 

The basic assumption of this theory is that in order to ensure bloating, the aggregate must show viscous behavior. As part of this viscous behavior, the pressure inside changes the pore structure inside. For acidic clay, it was confirmed that the formation of internal pressure is mainly caused by dehydration of crystalline water [19]. When the calcination time is short, because the aggregate reaches the foaming activation temperature before the dehydration of the crystalline water reaction is completed, the generated gas contributes to foaming by forming pressure inside. It was also found that the higher the bloating activation temperature, the lower the particle density. This occurs because as the sintering temperature increases, the amount of the liquid phase in the aggregate increases; accordingly, the viscous behavior of the aggregate increases. If the time is kept too short at the expansion activation temperature, it is not possible to provide the viscous behavior conditions for the agglomerate expansion, so an adequate holding time is required. It was found that the change in density was saturated at the holding time of 20 min or more.

**Figure 5:**
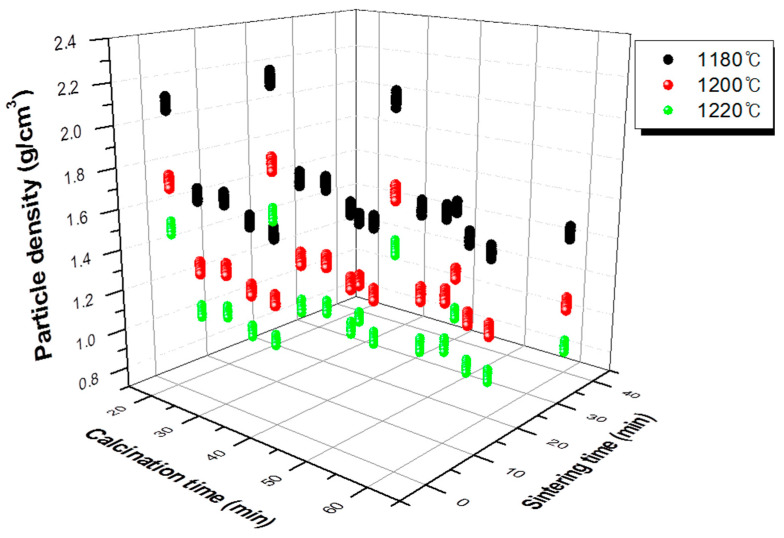
Expanded experiment results with the orthogonal array experiment design.

This result was expanded to 486 data instances, and the modeling of process variables was performed using this data. Figure 5 shows the results of data expansion through the orthogonal array experiment design.

### 5.2. Evaluation of the Model

The results of the three machine-learning models are summarized in Table 3. As shown in Table 3, for R^2^, SVR is the best model because this model is the largest and MSE, RMSE and MAE are the smallest. These values tend to improve with the number of data increases, given more data, this value will be improved. This means that a better model can be obtained by creating a model by expanding the experimental data than the modeling result using 18 data elements, which is the actual data. In fact, existing studies have reported that the creation of virtual data improves the R^2^ and MSE values of the model [47,48,49]. 

### 5.3. Experimental Evaluation of the Model Accuracy

**Figure 6:**
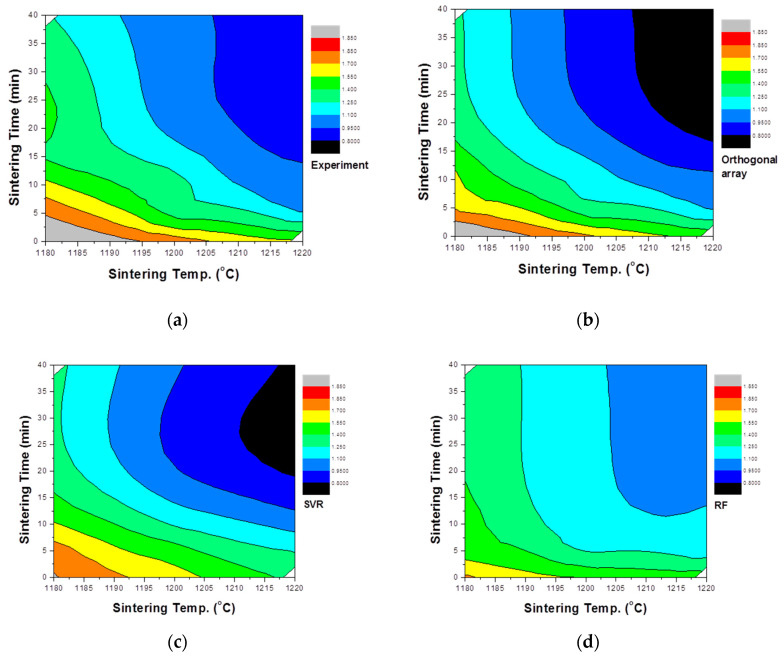
Particle density change of the aggregate with the soaking temperature and time as variables: (**a**) actual measurement, (**b**) orthogonal array-designed prediction, (**c**) SVR prediction, and (**d**) random forest prediction.

**Figure 7:**
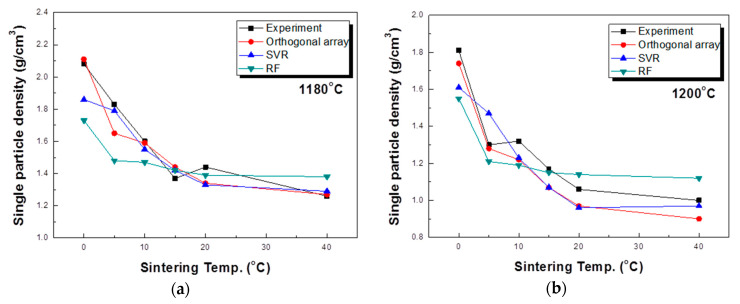

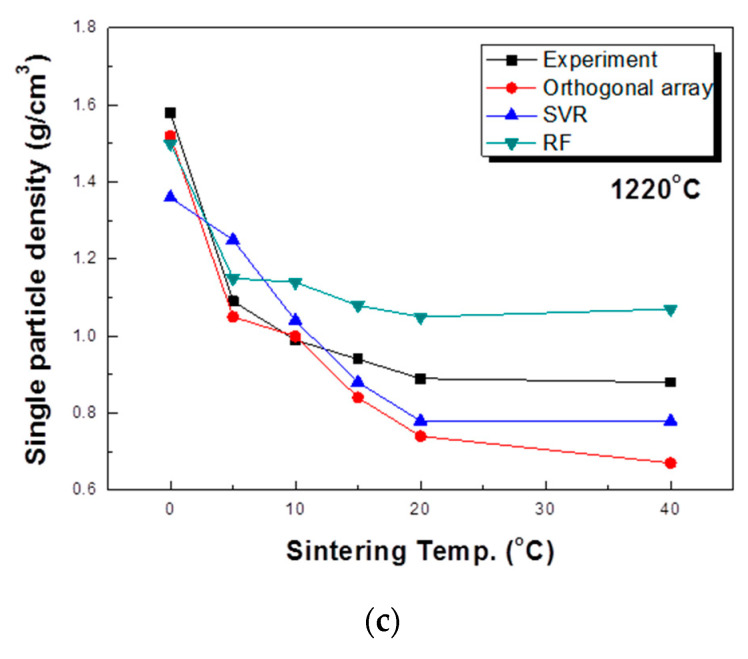
Prediction of the single particle density according to each analysis method: (**a**) 1180 °C, (**b**) 1200 °C, and (**c**) 1220 °C.

Additional experiments were conducted to compare the actual experimental results and modeling results. This is shown in Table 4. The main parameters, sintering temperature and sintering time, were considered, and calcination time and drying time were fixed. In order to compare the actual experimental results with the predicted trends for each model, contour lines were based on the measured density values with the sintering temperature and time used as variables (Figure 6). As a result of this analysis, the tendency for the density to decrease as the sintering temperature increases and with longer sintering times is clearly visible. The predicted value of the single particle density differed according to the modeling method. For the value predicted through the orthogonal array experiment design, the maximum value tended to be rather large and the minimum value rather small. This likely occurs because not all interactions are reflected when predicting values using the orthogonal array experiment design. For this reason, while orthogonal array experimental design techniques are used in many fields [50,51,52], their experimental predictions are not well utilized. In a study by Chen [53], an optimal process was designed using an orthogonal array table, but a loss function value was used instead of an experimental value. In this prediction result, as described above, the result was somewhat overestimated in terms of the maximum and minimum values. The results predicted by the random forest method were suitably reflected in the trends of the sintering temperature and time but were predicted to be more biased toward the average than the actual values. On the other hand, the result predicted by SVR was somewhat inaccurate in terms of the maximum value part compared to the actual experiment, but the properties were predicted to be closest to the most commonly measured result among the three models. For a better comparison of the actual experiments with the prediction results, the results of measuring the particle density of aggregates sintered at temperatures of 1180, 1200, and 1220 °C are compared with the predicted values of each model, as shown in Figure 7. The results predicted by the orthogonal array experiment design are in good agreement with the actual measurement results. However, the single particle density was predicted to be much lower than the measured values at 20 min and 40 min at a temperature of 1220 °C. For the random forest method, the coincidence of the trend was confirmed, but the single particle density was predicted to be relatively high at 20 and 40 min, representing the time points at which the single particle density change is nearly saturated. In the SVR case, the single particle density value at 0 min was predicted to be lower, but it was confirmed that the remainder was predicted to be closer to the actual experimental value. In particular, under the conditions of 20 min and 40 min at 1220 °C, the closest accurate predicted value was obtained. In general, in statistics, various methods are tried and verified when analyzing data because it is impossible to know which method is better before analysis. In this study, SVR appeared as the most suitable modeling method, and the reason is thought to be due to the characteristics of individual methods. Linear regression requires strong assumptions: linear relationship between dependent variables and the target variable, normally distributed errors, homoscedasticity of errors and independence of the points. In general, it is very hard to satisfy the above assumptions in real world data. Decision tree ensembles including random forest tend to be overly complicated in the process of learning non-linear relationships with a small number of points. As a result, it is likely to be unstable in the phase of prediction of new data. In this study, we use the kernel SVR with a radial basis function. Unlike the above two models, it is known that the kernel SVR is good at learning complex and non-linear relationships between variables [54].

**Figure 8:**
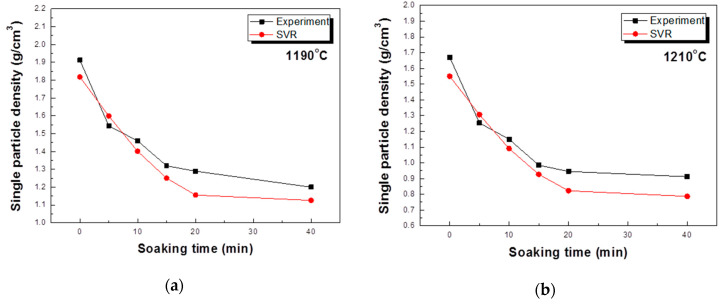
Prediction of the single particle density according to the SVR method: (**a**) 1190 °C, (**b**) 1210 °C.

In order to find out the predictive performance of the model for the unexperimented values, the physical properties were measured by firing the aggregate under conditions of 1190 °C and 1210 °C, and compared with the predicted values, and this is shown in Figure 8. At this time, the experimental conditions were fixed at r.t. ~300 °C and 300~600 °C for 60 min, and the condition at 600~bloating activation temperature was 20min. Most of the total predicted values were predicted close to the measured values. Experimental results with a sintering time of 0 to 15 min were quite consistent, and there was some error in the 20 to 40 min range, but the tendency to maintain the density was consistent. This means that modeling results using SVR can be predicted even in unexperimented areas, and the results are also suitable. In the expansion of data by an orthogonal sequence table, the results of the tested variables can be known, but the untested variables could not be defined.

## 6. Conclusions

In this study, the experimental design of the change of particle density according to drying, calcination, and sintering conditions of artificial lightweight aggregate were performed using an orthogonal array table, and data were expanded. Modeling using machine-learning techniques were performed using the expanded data, and the following conclusions were drawn about the effectiveness of data expansion by an orthogonal array table and the appropriate modeling method:

1. The experimental design using the orthogonal array table and the expanded data gained through this method did not show a significant difference from the measured values.
2. The SVR model showed the best prediction accuracy among the reviewed models and generally showed good results even with untested variables.
3. Modeling by the random forest method predicts the trend of the process well. However, the result was predicted to be closer to the mean value than the actual value, and it could not be predicted for an untested part.
4. Through the experimental design and the expansion of the data by the orthogonal array table and modeling with the machine-learning technique, a model capable of efficiently predicting physical properties was realized.

In this experiment, only 18 data elements were actually tested for modeling, but these can be expanded to 486 through an orthogonal array table. This is a small amount of data in the field of data science but sufficient in the modeling field of scientific experiments. This means that the expansion of data through an orthogonal array table is useful for actual modeling. If there is a more considerable amount of data, the accuracy of the model can be improved. Hence, it is important to attempt this with more data. Orthogonal array experimental designs can be used to expand the number of variables, and in upcoming studies we will design experiments involving more process variables to evaluate the adequacy of the model. Also, studies on the correction of extended data and improvement of model reliability will be conducted.

## Figures and Tables

**Table 1:** Processing variables by item.

Process	Process Temp. (°C)	Variables	
Drying and preheating	25 ~ 300 °C	20, 40, 60 min	
	300 ~ 600 °C	20, 40, 60 min	
Calcination	600 °C ~ B.S.A. temp.	20, 40, 60 min	
Bloating Start and Activation (B.S.A.)	1180, 1200, 1220 °C	Time	0, 5, 10, 15, 20, 40 min

**Table 2:** Orthogonal array experimental design and the measured density.

No.	Firing Condition					Single Particle Density (g/cm^3^) (mean)
	Sintering Time	B. S. A. Temp. (°C)	600 ~ B.S.A. Temp. Process Time (min)	300~600 °C Process Time (min)	r.t. ~ 300 °C Process Time (min)	
1	0	1180	20	20	20	2.09
2	0	1200	40	40	40	2.03
3	0	1220	60	60	60	1.76
4	5	1180	20	40	40	1.72
5	5	1200	40	60	60	1.49
6	5	1220	60	20	20	1.27
7	10	1180	40	20	60	1.84
8	10	1200	60	40	20	1.5
9	10	1220	20	60	40	0.98
10	15	1180	60	60	40	1.73
11	15	1200	20	20	60	1.13
12	15	1220	40	40	20	1
13	20	1180	40	60	20	1.57
14	20	1200	60	20	40	1.1
15	20	1220	20	40	60	0.88
16	40	1180	60	40	60	1.46
17	40	1200	20	60	20	0.94
18	40	1220	40	20	40	0.95

**Table 3:** Modeling performance.

	Linear Regression	Random Forest	SVR
$R^{2}$	0.799	0.783	0.933
MSE	0.029	0.031	0.009
RMSE	0.171	0.178	0.098
MAE	0.146	0.143	0.071

**Table 4:** Additional experimental conditions for model verification.

Process	Process Temp. (°C)	Variables	
Drying and preheating	25~300 °C	60 min Fixed	
	300~600 °C	60 min Fixed	
Calcination	600 °C ~ B.S.A. temp.	20 min Fixed	
Bloating Start and Activation (B.S.A.)	1180, 1190, 1200, 1210, 1220 °C	Time	0, 5, 10, 15, 20, 40 min
